# Smoking in school-aged adolescents: design of a social network survey in six European countries

**DOI:** 10.1186/s13104-015-1041-z

**Published:** 2015-03-21

**Authors:** Vincent Lorant, Victoria Eugenia Soto, Joana Alves, Bruno Federico, Jaana Kinnunen, Mirte Kuipers, Irene Moor, Julian Perelman, Matthias Richter, Arja Rimpelä, Pierre-Olivier Robert, Gaetano Roscillo, Anton Kunst

**Affiliations:** Institute of Health and Society, Université Catholique de Louvain (UCL), Clos chapelle aux champs 30.05, 1200 Brussels, Belgium; Escola Nacional de Saúde Pública, Universidade Nova de Lisboa, Lisbon, Portugal; Department of Human Sciences, Society and Health, University of Cassino and Southern Lazio, Cassino, Italy; School of Health Sciences, University of Tampere, Tampere, Finland; Department of Public Health, Academic Medical Center, University of Amsterdam, Amsterdam, The Netherlands; Institute of Medical Sociology, Martin-Luther University of Halle, Halle, Germany; Department of Adolescent Psychiatry, Tampere University Hospital, Tampere, Finland

**Keywords:** Smoking, Inequalities, Social network, Schools, International comparison

## Abstract

**Background:**

In Western countries, smoking accounts for a large share of socio-economic inequalities in health. As smoking initiation occurs around the age of 13, it is likely that school context and social networks at school play a role in the origin of such inequalities. So far, there has been little generic explanation of how social ties at school contribute to socio-economic inequalities in smoking. The SILNE (*Smoking Inequalities – Learning from Natural Experiments*) survey was designed to test the hypothesis that a combination of peer effect, homophilous social ties, and school context may explain how smoking inequalities are magnified at school – a theory known as network-induced inequality. In this paper, the survey theory and design are presented.

**Findings:**

The social network survey was carried out in 2013 in six medium-sized European cities with average incomes similar to the national average: Namur (Belgium), Tampere (Finland), Hannover (Germany), Latina (Italy), Amersfoort (The Netherlands), and Coimbra (Portugal). In each city, 6 to 8 schools were selected in a stratified sampling procedure. In each school, two grades in secondary education, corresponding to 14-16-year-olds, were selected. All adolescents in these two grades were invited to participate in the survey. Social ties were reported using the roster approach, in which each adolescent had to nominate up to 5 friends from a directory.

The survey collected information from 11,015 adolescents in 50 schools, out of a total of 13,870 registered adolescents, yielding a participation rate of 79%. The SILNE survey yielded 57,094 social ties, 86.7% of which referred to friends who also participated in the survey.

**Discussion:**

The SILNE survey was designed to measure the association between adolescents’ social ties at school, their socio-economic background, and their smoking behaviour. Two difficulties were encountered, however: legal privacy constraints made it impossible to apply the same parental consent procedure in all countries, leading to somewhat lower participation rates in two cities: Hannover and Latina. It was also difficult to match the 6 cities in terms of both age and type of education.

The SILNE survey provided a comparable database for the study of smoking inequalities across European cities from a social network perspective.

**Electronic supplementary material:**

The online version of this article (doi:10.1186/s13104-015-1041-z) contains supplementary material, which is available to authorized users.

## Background

Worldwide, smoking is a major contributor to the burden of disease, accounting for 6.3% of the total burden [1]. In addition, smoking is the leading behavioural contributor to socio-economic inequalities in health [2,3]. Recent trends in the analysis of smoking inequalities suggest that inequalities have not decreased and may even have increased in some countries [4,5]. As most smokers begin smoking around the age of 13, understanding smoking inequalities in adolescence is an important step to explaining smoking-related mortality across social strata in adulthood [6]. The project “Tackling socio-economic inequalities in smoking: learning from natural experiments by time trend analyses and cross-national comparisons” - SILNE implemented a social network survey that aimed to analyse how social ties at school influence socio-economic inequalities in smoking and how such influences depend on the school context. This paper begins with a brief overview of socio-economic inequalities in smoking among adolescents, before presenting the survey's rationale and design.

### Smoking: socio-economic inequalities among adolescents: an overview

In several Western countries, adolescent smoking is more widespread in lower socio-economic groups than in higher socio-economic groups [7-11]. For example, higher parental educational status was a significant protective factor in the Avon Longitudinal Study, in the UK: 13-year-old children whose parents had a higher educational level were less likely to smoke than children whose parents had a low educational level. However, not all studies display the same pattern. As acknowledged in a review, low socio-economic status is generally but not ubiquitously associated with smoking [11]. The review, which compared socio-economic differences in smoking behaviour among young people, identified 21 high-quality, nationally representative studies of smoking prevalence among adolescents in Western countries. It found “some support” for a higher prevalence of smoking among low socio-economic groups of adolescents compared with high socio-economic groups of adolescents [12]: 9 studies reported more frequent smoking in lower socio-economic groups compared with higher socio-economic groups; one study reported the opposite; 6 studies showed no significant association; and the remaining studies had mixed results. A recent empirical study comparing the association between family affluence and adolescent smoking in 33 European countries, Israel, and Canada identified two patterns: the prevalence of adolescent smoking was higher in less affluent countries, but the difference in smoking prevalence between socio-economic groups was greater in more affluent countries [13].

In addition, these inequalities may be increasing over time: a Finnish study of smoking inequalities among adolescents measured by familial SES between 1991 and 2007 concluded that there “are persistent or even increasing socioeconomic differences” [14]. In Germany, too, smoking inequality, measured according to the family affluence scale, did not decrease between 1994 and 2002 [15]. The latter study highlighted the importance of the type of school as an indicator of smoking inequalities. Family affluence was a smaller risk factor for smoking, compared with the risk attached to the type of school: the type of school and individual social position were more important predictors of smoking behaviour than parental SES, although family affluence and type of school are generally interdependent [14,15].

### Theory of network-induced inequality

These findings on smoking inequalities show the need for a better understanding of the association between smoking and socio-economic status and of how such inequalities might emerge among school-age adolescents. The SILNE survey aimed to investigate the role of school-based social networks in magnifying these inequalities. The role of schools in explaining smoking inequalities has been hinted at in previous studies, in which school SES was related to smoking inequalities [10,15-17].

The SILNE survey postulated that schools may contribute to smoking inequalities through three components: friendship ties, homophily, and school context. First, friendship ties at school are a possible major driver of smoking initiation and maintenance, through a mechanism known as peer effect ([18] p.44). Several recent social network surveys have shown that smokers are more likely to befriend smokers and conversely for non-smokers [19-22]. For example, the Add Health study found that the odds of starting smoking increased by 1.8 for each smoking friend among an adolescent’s friends [23]. Similar results were found in Finland [24], in the European ESFA study [20,25], in the ASSIST programme study in Wales and England [19], and in the North Carolina study [26]. These network studies, however, have not clarified how social relationships may become drivers of socio-economic inequalities in adolescent smoking behaviour. Second, adolescent social ties, like those of adults, are homophilous: adolescents befriend adolescents of a similar socio-economic background [27], leading to enhanced clustering of smoking among adolescents of similar socio-economic background. Third, this kind of peer effect and homophily may be sensitive to school context [28]: educational systems may allocate adolescents to different kinds of education according to their academic skills, leading to less social mixing either between schools or within schools. This kind of differentiation has been shown to be associated with higher inequalities in educational performance [29] and also, in some studies, in smoking [30,31]. The SILNE survey hypothesised that the combination of peer effect and homophily might help to explain how smoking inequalities are magnified at school, a theory known as network-induced inequality [32,33]. So far, adolescent surveys, such as the Health Behaviour in School-aged Children (HBSC) survey or the European School Survey Project on Alcohol and Other Drugs (ESPAD), have not directly described adolescent social ties at school. In addition, cross-comparative social network surveys in European schools are rather rare. SILNE was one of the first international social network surveys of adolescents at school across different European contexts to focus on the details of adolescent smoking behaviour.

In this paper, the SILNE survey protocol and its sampling design are presented. This protocol describes a design and a questionnaire for collecting and analysing the effect of social relationships on socio-economic inequalities in smoking and some other health behaviours across different European contexts.

## Findings

### Setting and participants

The survey was carried out between January and November 2013 in one city each in six European countries. The six selected countries are diverse in three ways. First, three countries have high differentiation in school tracks at an early age, based on academic achievement (Belgium, Germany, and the Netherlands); two countries have lower differentiation (Italy and Portugal) [34,35]; in one country (Finland), there is no tracking, yet, in the age groups covered by the study. Second, the PISA Sciences study shows that the first group of countries also displays stronger associations between adolescents’ performance and their social, economic, and cultural status than the latter set of countries [36]. Third, Finland and Belgium score high on the tobacco control scale (TCS) (scores of respectively 52 and 50 out of 100), while Italy and the Netherlands have medium values (TCS of 47 and 46, respectively) and Portugal and Germany have the lowest scores (TCS, respectively, of 43 and 37) [37].

In each country, a city was selected in which population size, income, and employment rates were all close to the national average. The cities of Namur (Belgium), Tampere (Finland), Latina (Italy), Amersfoort (Netherlands), Hannover (Germany), and Coimbra (Portugal) were selected. These cities are described in Table 1 (see Additional file 1: references Table 1). In each city, schools were selected from the local register of schools, from two strata, and were approached to participate in the SILNE survey. School stratification was achieved according to information specific to each country: either by the type of school (Italy, Germany, and the Netherlands), by the socio-economic ranking of the school by the educational authorities (Belgium and Portugal), or according to the area’s socio-economic status (Finland) (Table 2).

**Table 1:** **SILNE 2013 Survey: socio-economic characteristics of the cities surveyed in the 6 countries**

**Country**	**City**	**Description of the city**	**Average income of country (€ )**	**Average income of the region (or province) where the city is located (€ )**	**Unemployment rate in the country**	**Unemployment rate in the city**	**Population**
Belgium	Namur	Namur is the capital both of the province of Namur and of Wallonia. The city is an important commercial and industrial centre. It produces machinery, leather goods, metals, and porcelain.	€18,301	€18,785	7.6%	12.6%	108,950
Finland	Tampere	Tampere is the third-largest city in Finland. It is a centre of leading-edge technology, research, education, culture, sports, and business.	€25,500	€25,000	9.7%	12.8%	215,168
Germany	Hannover	Hannover is the capital of the federal state of Lower Saxony and is a major centre in northern Germany, known for hosting annual commercial trade fairs such as the Hanover Fair and CeBIT.	€30,360	€34,308	8.1%	9.2%	514,137
Italy	Latina	Latina is the capital of the province of Latina in the Lazio region. The city has some pharmaceutical and chemical industry and is an important centre for agriculture.	€22,891	€20,487	8.4%	10.6%	118612
Netherlands	Amersfoort	Amersfoort is the second-largest city of the province of Utrecht. Amersfoort is one of the largest railway junctions in the country.	€23,400	€24,900	6.4%	6.4%	148,250
Portugal	Coimbra	Coimbra is the main city of the Centre Region. Its main activities are in the fields of commerce, public administration, education, health, and social services.	€12,408	€12,348	12.7%	10.0%	143,396

Sources: see Additional file 1 – references Table S1.

**Table 2:** **SILNE 2013 Survey: grade and school selection, ethical approval, sample and participation rate per city**

**City (Country)**	**Selected grades**	**Criterion of school selection and stratification of schools**	**Name generator**	**Consent**	**No. of schools invited**	**No. of schools recruited**	**School staff recruited**	**No. of registered adolescents**	**No. of adolescents participating**	**Participation rate %**
Namur (BE)	Last grade of lower secondary and first grade of upper secondary education (3rd and 4th) Early educational tracking (at the age of 12)	Selection and stratification based on the parental socio-economic status index (low SES = index < =14 and High index >14)	Roster	Passive for parents; active for adolescent	21	7	85	2,375	2,133	89.8
Tampere (FI)	Last two grades of lower secondary education (8th and 9th). Later educational tracking (at the age of 16)	Selection and stratification of low and high SES based on the average income in the area, proportion of highly educated people in the area (of over-15-year-olds), and distance from the school to the centre of Tampere.	Name generator	Passive for both	14	8	32	1,744	1,499	86.0
Hannover (DE)	Last two grades of lower secondary education (8th and 9th) Early educational tracking (at the age of 10)	Selection and stratification of low and high SES based on the average income in the area and the tracking/school types (low, mixed, medium, and high educational tracks).	Roster	Active for both	64	13	67	2,238	1,476	66.0
Latina (IT)	First two grades of upper secondary education (9th and 10th) Later educational tracking (at the age of 14)	Selection and stratification of low and high SES based on school type: vocational and high schools.	Roster	Active for both	12	8	36	2,727	2,085	76.5
Amersfoort (NL)	Last two grades of lower secondary education (3rd and 4th) Early educational tracking (at the age of 12)	Selection and stratification of low and high SES based on the available school tracks in the school: vocational level, middle, and high level.	Roster	Passive for both	38	8	28	2,377	1,922	80.9
Coimbra (PT)	First two grades of upper secondary education (10th and 11th) Later educational tracking (at the age of 15)	Selection and stratification of low and high SES based on the average income in the area and school type: vocational and general schools.	Roster	Passive for both, but one school	14	6	25	2,409	1,900	78.9
Total					163	50	273	13,870	11,015	79.4

To motivate school principals to participate, each school was promised a school feedback in which results for the school would be compared to the others schools participating within the city and/or the European average (see supplementary material, with examples, at http://silne.ensp.org/instruments_wp5/). After they agreed to participate, leaflets and detailed information were sent to schools, teachers, parents, and students according to each country’s national regulations. The leaflets explained the objective of the research, the research team at national and international level, the topics addressed in the questionnaire, the benefits of participation for schools, the confidentiality of the adolescents’ answers, voluntary nature of participation, and adolescent and/or parental consent (see supplementary material at http://silne.ensp.org/instruments_wp5/).

In each school, two groups of school years were selected, corresponding to adolescents aged between 14 and 16. This is the group most relevant for the transition to weekly smoking [38]. This age group corresponded to the last two grades of lower secondary education in Finland, Germany, and the Netherlands, to the first two grades of upper secondary education in Italy and Portugal (Table 2), and to the last grade of lower secondary and the first grade of upper secondary in Belgium. In Portugal, the organisation of the educational system made it difficult to match exactly the same age group as was covered in the other schools. In those two grades, all registered adolescents were invited to participate in the survey.

### Design and instruments

In each school, SILNE applied a whole-network approach [39,40], with the network defined as all adolescents registered in the two selected grades. The survey had two instruments: an adolescent questionnaire and a school-staff questionnaire. The adolescent questionnaire was divided into several main sections: social ties, smoking, other health risk behaviours, socio-demographics, and school climate (see Table 3).

**Table 3:** **SILNE 2013 survey: topic, concepts, and instruments**

**Topic**	**Concepts**	**Instrument question number**
**Adolescent questionnaire**		
Social ties	Cooperative relationships	
	Classmates whom respondent prefers to work with or ask for advice, for example on homework or on an assignment [49,50]	B
	Friendship relationships	
	Your best friends in two grades [50]	C
Demographics	Immigration status [51,52]	
	Birth country	Q3
	Birth country of parents	Q34, Q35
	Years living in host country	Q4
	Language spoken at home	Q43
Lifestyle and health	Self-reported health [53]	Q5
	Long-term illness [53]	Q6
	Physical activity [54]	Q7
	Consumption of alcohol [54]	Q9
	Use of cannabis [54]	Q12
Smoking	Smoking status [54]	
	Ever tried smoking	Q13, Q15, Q16
	Regular smoking	
	Daily smoker	
	Smoking dependence index	
	Fagerström Tolerance Questionnaire [55]	Q19, Q20, Q21, Q22
	Onset of smoking [54]	Q14
	Where and when adolescents smoke [56,57]	Q23
	Access to cigarettes [54]	Q25
	Injunctive social norms	
	Perception of parents’ and friends’ approval and disapproval of smoking/the intention to smoke [58-60]	Q26, Q27, Q32, Q33
Smoking	Peers’ smoking	
by peers and parents	Number of close friends who smoke [61]	Q33
	Parents’ smoking	
	Members of the household who smoke [61]	Q51
Smoking rules and perceptions		
	Smoking rules at home [54]	Q52
	Enforcement of tobacco control policies at school [62]	Q61
	Perception of smoking behaviour at school: adolescents and teachers [63]	Q58, Q59, Q60
Household	Structure of the family	
	Members of primary adolescent’s household [52]	Q42
Socio-economic status	Parents’ occupational level [52]	Q38, Q40
	Parents’ educational level [52]	Q36, Q37
	Family Affluence Assessment scale [64]	Q44, Q45, Q46, Q47
	Subjective socio-economic status of household	
	Adapted Mc Arthur Scale – SES ladder – Youth version [46]	Q49
	Adolescent’s weekly disposable income [54]	Q50
School context	Academic performance [54]	Q54
	School Engagement Assessment [65]	Q55
	School Burnout Inventory [66]	Q56
	School connectedness/school climate [54]	Q57
Perception of risk of behaviour	Perception that smoking cigarettes is harmful to your health [67]	Q62
	Health expectations [68]	Q64
**School questionnaire**		
School size	Number of students, teachers, and classes [54]	SW1
Physical school environment	Facilities in the school and in school neighbourhood [52]	SW5, SQ1, SQ2
	Neighbourhood characteristics of schools [52]	SW6
	Perception of social climate at school [52]	SW7
School smoking policies	Adoption of policy prohibiting cigarette smoking: when, where, and for whom [54]	SQ5, SQ6, SQ7, SQ8,
	Consequences for breaches of smoking rules by adolescents [69]	SQ15
	Information on smoking rules at school [69]	SQ13, SQ16, SQ17, SQ18
Health promotion and prevention programmes	Health promotion activities by schools [69]	SQ19, SQ20, S21
	Tobacco use prevention training for school staff [69]	SQ25

The social ties name generator was developed on the basis of the previous Add Health survey [41]. As recommended in the literature [42-44], two name generators were included, with one related to instrumental ties involving cooperation (“whom do you prefer to work with?”) and the other related to friendship ties (“who are your best and closest friends?”). For each name generator, adolescents were asked to nominate up to 5 alters (schoolmates or friends) in the two grades that were included in the study. For the second name generator, adolescents were also asked to nominate whom they met after school and whom they talked to on the phone or chatted with on Facebook. For every nomination, the adolescents were also asked to state whether this adolescent became their classmate or friend that year or before.

To answer the social ties questions, adolescents were handed a directory. This directory contained the first names and family names of all adolescents enrolled in the two grades in question. The names were listed alphabetically by class and grade. One primary code was assigned to each name and respondents were asked to write the corresponding codes into their questionnaire (a roster approach). In Finland, the researchers were not allowed access to the list of adolescents enrolled in the school and therefore the names of adolescents had to be written on the questionnaire and the research fellows coded them afterwards (name generator approach). When answering, the adolescents could nominate any alter in other classes: the literature has indeed shown that a significant proportion of ties are outside the class, that classes tend to be reshuffled over time, and that students repeating a year keep ties with their previous grade.

The adolescent questionnaire included questions on health (self-rated health and long-term illness), health expectations, and health behaviour (physical activity, drinking, and cannabis use). Questions about smoking included smoking initiation, smoking frequency and quantity, nicotine dependence, where and when the adolescent smoked, and where he or she accessed cigarettes. In addition, the survey asked about descriptive and injunctive smoking norms, smoking by family members, smoking rules at school, and smoking rules at home. Socio-economic status was measured by a combination of objective and subjective indicators, with information on both parents and on the individual concerned: the Family Affluence Scale of Health Behaviour in School-aged Children (HBSC) survey [45], the McArthur Scale of subjective social status – youth version [46], and the mother’s and father’s educational level (low, middle, or high, according to ISCED) and employment status (employed, unemployed, or inactive). In addition, adolescents were asked to state how much pocket money they received or earned for themselves.

As previous multi-level studies have shown that the school context may influence smoking behaviour [47,48], adolescents were requested to rate the school climate, the risk of burnout at school, and their academic engagement and achievement. The adolescent questionnaire was translated into local languages from an original written English version and was piloted in one school within each country. A few questions were omitted from the questionnaire due to the high rate of “no use” responses (e.g. to the question about chewing tobacco, to which the 96.2% of adolescents answered “never ever tried”) and other questions were reformulated in order to make them more understandable. The schools where the questionnaire was piloted were excluded from the sample of schools chosen to carry out the survey afterwards and their data were deleted.

The school-staff questionnaire was filled in by educational staff, including teachers, or by the principal. This questionnaire was divided into four sections: the physical school environment, smoking control policies, health promotion and prevention, and demographic information on the respondent. Most of the questions were adopted from HBSC, Youth Smoking Survey (YSS), and School Health Policies surveys (SHPSS) carried out by the Centers of Disease Control and Prevention (CDC), which have been widely used.

The survey administration was by paper and pencil (see both adolescent and school questionnaires in the supplementary online material). Questionnaires were administered in class rooms during school hours by one or two researchers and, in some cases, with the help of a teacher. After the research objectives were explained, the adolescents were requested to participate and received the school directory and the questionnaire, which, on average, took 30 minutes to fill in.

### Ethical issues

In each country, ethical approval from local or national ethical committees was requested and obtained (see Additional file 2: Ethical information). In addition, in some countries, permission to conduct the survey was requested from educational authorities. School principals, parents, and adolescents received leaflets, information letters, and parental consent letters, according to each country’s regulations (see Table 2). Active parental consent was required in Italy and Germany. To protect adolescents’ privacy, two procedures were applied. First, two different individuals managed the questionnaires and the school directories. These two individuals were legally committed not to link the information in the two kinds of document and to manage the two kinds of material separately. Second, two name-blinding procedures were applied. Firstly, the adolescents had to report in their questionnaire the code of the nominees and not the names (with the exception of Finland). Secondly, primary codes were replaced by random codes by a Trusted Third Party (TTP) from the IT Security Management of the University of Louvain, which is legally committed to protect privacy. This TTP is under the authority of the rector of the University and is not accountable to the SILNE researchers. All these procedures were declared to the Belgian Privacy Commission (decision No. 1350057189088) and approved by the Ethical Committee decision No. 2012/09oct/461.

### Sample size and weighting

The sampling design aimed to provide information on both individuals and networks. In each city, the survey aimed to collect questionnaires from about 1,800 adolescents from 6 to 8 secondary schools stratified according to the school type or to the school’s socio-economic status (SES). The sampling size was computed to estimate a regular smoking proportion difference of 15% (high SES) to 20% (low SES), with alpha = 5% and Beta =20%.

As all adolescents of two grades in the participating schools were recruited, there was no need to take into account school size in the weighting: bigger schools contributed more observations than smaller schools. However, as the participation rate may differ across schools, for population inference, data were weighted by the inverse of the participation rate according to the formula below, where N_ij_ is the registered number of adolescents in city i, school j, and n_ij_ is the number of adolescents who actually participated in the survey. Our results were weighted accordingly.

$$ {w}_{ij}=\frac{N_{ij}}{n_{ij}}\;\frac{{\displaystyle {\sum}_j{n}_{ij}}}{{\displaystyle {\sum}_j{n}_{ij}}} $$

### Data management

After the survey was carried out, the data entry was centralised, using a Web-based platform. In each country, a unique password was created to allow those responsible for data entry to use the platform. The identification codes (country, school, and adolescent codes) were added to the platform in order to facilitate the process of entering data and to reduce double coding or typing errors in the entering of adolescent codes (i.e. in answers to social network questions). More than one person was allowed to enter data at the same time. Guides to utilisation of the web-based platform and constant e-mail support were available. When the data entry phase was over, data quality was centrally assessed by the European coordinator of the survey. The consistency of the data was reviewed by checking all questionnaires with high number of unanswered questions, completing individual characteristics where possible, and checking inexistent codes. Given the negative and exponential consequences of omitting any questionnaire from the sociometric data, no questionnaire was discarded from the database. An indicator variable was created in in the database, reporting adolescent questionnaires with more than 20 missing variables; this was the case with 45% of the questionnaires. Database files including school, school-staff, and adolescent socio-economic and social ties data in SPSS, Stata, SAS, and text files were sent to the researchers one month after the data entry phase was over.

### Results of the survey

Fifty schools out of the 163 contacted participated in the survey. The number of schools varied by country, with a minimum of 6 schools (in Portugal) and a maximum of 13 (in Germany) (Table 2). The schools participating were more frequently from low SES (n = 31) than high SES (n = 19). Broadly, the distribution of low SES and high SES schools was balanced within each country, with the exception of Portugal (2 high-SES schools *vs* 4 low-SES) and Italy (2 high-SES schools *vs* 6 low-SES schools). Portugal had a larger average school size than Germany. The number of classes ranged from 87 in Germany to 154 in Belgium. The participating schools had a combined total of 13,870 registered adolescents in the two grades selected. 11,015 adolescents participated in the survey, yielding an average participation rate of 79.4%. The non-participants were classified into three categories: adolescents absent on the days of the survey (n = 1864), adolescents unwilling to participate (n = 461), and others whose questionnaires were discarded because they were blank or obviously incoherent (n = 65, a minimum of 2 in the Netherlands and a maximum of 17 in Italy). In addition to the adolescent questionnaire, the researchers were able to collect 273 questionnaires from school staff, with an average of 5.5 questionnaires per school.

The reasons why some schools were not willing to participate were quite diverse, but the most frequent reasons were that the survey timing was not compatible with their scheduled school activities, that they were already participating in other surveys or research activities, or that they were not interested in the survey topic. Concerns about privacy were raised by a handful of schools too. The researchers found that the following elements played a positive role in recruiting schools in some countries: already being in contact with the schools, having a personal contact with the principal and/or visiting the schools to make arrangements, the offer of feedback to the school on its own results with a comparison to the average of the 6 cities, anticipating and considering school planning of activities, having documents in each country’s language to explain the research project, and getting official support from local educational authorities.

The fieldwork was carried out without major problems. The main challenges were related to the use of the directory: new adolescents not being included in the school directory (0.4%) or some adolescents not writing down their assigned code in the questionnaire (1.4%). Other concerns were related to answering adolescents’ requests for clarification, ensuring that adolescents did not speak to each other when filling in the questionnaire, especially when they were using the directory to answer social network questions, ensuring that teachers did not interfere with adolescents’ answers, and coping with adolescents who had completed the questionnaire early (particularly the non-smokers). In addition, sometimes schools had to be visited more than once when a parental consent form was required before adolescents could fill in the questionnaire.

The overall rate of missing information was low. Only 111 adolescents did not answer the question about “ever trying smoking a cigarette”, 44 adolescents did not answer any of the questions in the section of the questionnaire about smoking, and 215 adolescents did not follow the question sequence to answer correctly the questions related to smoking.

The adolescents had a mean age of 15 years; more females than males participated (Table 4). According to family affluence scale and parents’ education, the socio-economic status was highest in Finland and the Netherlands and was lowest in Italy and Portugal. The average prevalence of daily smoking among adolescents in all countries was 17.4%, with higher prevalence in Latina (26.8%) and Namur (24.5%) and lower in Hannover (12.2%) and Amersfoort (12.4%).

**Table 4:** **SILNE 2013 Survey, socio-demographics and prevalence of smoking: number and percentage per city**

**European Cities**	**Age**			**Gender - female**		**Family Affluence Scale FAS (Scale 0–7)**			**Low level of parents’ education**		**N**	**Daily smokers**	
									**Father**	**Mother**			
	**Mean**	**Std**	**N**	**%**	**N**	***Mean***	**Std**	**N**	**%**	**%**		**%**	**N**
Namur (BE)	15.6	1.1	2,118	50.5	2,099	5.3	1.2	2,133	16.9	13.0	2,133	24.5	2,133
Tampere (FI)	14.8	0.7	1,497	49.4	1,499	5.5	1.6	1,499	4.26	2.7	1,499	16.8	1,499
Hannover (DE)	14.7	0.9	1,439	50.4	1,438	5.2	1.2	1,476	13.0	12.4	1,476	12.2	1,476
Latina (IT)	15.2	1.0	2,081	57.9	2,083	5.0	0.8	2,085	34.4	29.4	2,085	26.8	2,085
Amersfoort (NT)	15.0	0.9	1,914	50.3	1,913	5.9	1.1	1,922	14.7	15.0	1,922	12.4	1,922
Coimbra (PT)	15.9	1.0	1,885	50.0	1,900	5.6	1.2	1,900	37.2	28.9	1,900	19.7	1,900
All Cities	15.2	1.1	10,934	50.7	10,932	5.5	1.4	11,015	18.6	15.9	11,015	17.4	11,015

SILNE 2013 figures are weighted according to adolescent participation rates in schools.

The SILNE survey yielded 57,094 social ties within the two grades (all classes from the directory), 86.7% of which referred to an alter participating in the survey. As the percentage is higher than the overall participation rate (79.4%), this indicates that non-participating adolescents have, on average, fewer ties than participating adolescents: they could be newcomers, be more likely to be absent, or be less popular or less socially active. Responses to the social networks questions in the survey were higher in Portugal, Belgium, and Finland and somewhat lower in Germany (Table 5).

**Table 5:** **SILNE 2013 Survey: number of friendship nominations per country and participation status**

**Citation Status**	**Namur-Belgium**		**Tampere-Finland**		**Hannover-Germany**		**Latina-Italy**		**Amersfoort-Netherlands**		**Coimbra-Portugal**		**All cities**	
	**Ties**	**%**	**Ties**	**%**	**Ties**	**%**	**Ties**	**%**	**Ties**	**%**	**Ties**	**%**	**Ties**	**%**
Nominated, not participating	1,196	10.8	745	10.7	1,582	21.2	1,761	15.1	1,345	13	991	10	7,620	13.3
Nominated, participating	9,916	89.2	6,195	89.3	5,880	78.8	9,915	84.9	9,021	87	8,547	90	49,474	86.7
Total	11,112	100	6,940	100	7,462	100	11,676	100	10,366	100	9,538	100	57,094	100

## Discussion

The fieldwork for the SILNE survey yielded a number of interesting lessons for further international social network surveys in relation to adolescent health. First, it was possible to apply the same method across six different European cities, although slight differences were unavoidable. One possibility would be to improve network size comparability: the researchers found that the school size in Germany was somewhat smaller than in the other countries. Future comparative studies of school networks could be stratified not only according to school socio-economic status (this was SILNE’s key objective) but also according to school size, an important determinant of the network structure. Another improvement would be to better match the age group and network borders, by surveying the whole school instead of selecting two grades.

Legal privacy constraints made it impossible to apply passive consent in two countries, leading possibly to somewhat lower participation rates. For some schools surveyed in Germany and Italy, either teachers did not remind adolescents to bring back their consent forms or adolescents did not bother to bring them, even when they had been signed by their parents. Other studies have shown the same results: in the schools that participated in the Add Health study and asked for active consent, the participation rate was extremely low, ranging from 20% to 60% [49]. Thus, when the survey administration requires using active consent, additional visits to schools prior to the survey should be scheduled in order to remind school staff and teachers, as well as adolescents, to bring in their consent forms.

Another difference in the fieldwork carried out between cities was the use of a name generator in Finland and a roster in the other countries. Requesting the list of adolescents enrolled in two grades from school principals meant spending more time and effort in approaching schools and convincing them to participate in the study. The researchers used leaflets, letters, and phone calls and arranged more than one visit to schools in order to explain the need for the adolescent directory, the security system created to manage it, and its confidentiality and blinding procedures. In all countries, except Finland, these arguments favoured a roster over the name generator. Further analysis will be required to test whether these two differences affected the results.

Providing principals with individualised school feedback was in most countries an effective way of motivating schools. Several issues were addressed in the feedback in addition to smoking: social ties, health behaviour, and school connectedness, as well as some guidance for health promotion. This feedback offered schools a clear signal of the benefits they would enjoy as a result of participation and helped them cope with their legal obligations (in most cities) in health promotion.

A further difficulty was to match the countries in both age and type of education. Age is a key risk factor for smoking, but so is the type of education (vocational vs academic orientation). As educational systems differ across countries, it was not possible to select both the same age group and the same educational track in all six cities. The researchers selected the grades corresponding best to the 14–16 age group. This age group corresponds to the last two grades of “lower education” in 4 countries, but to the first two grades of “upper education” in Italy. In Portugal, 14 year-old adolescents and 15+ adolescents are taught in different types of education and schools. Our target age group overlapped two different types of education, covered by different schools. As a result, in Portugal a slightly older age group was surveyed. Statistical analysis will thus need to control for these age group differences.

International social network analysis needs to combine two objectives: having the same age group, to allow for health behaviour comparison across countries, and having the same definition of network borders (school grades), to allow for comparison of networks. Our study shows that these two objectives may conflict with each other because education systems differ.

## Conclusions

SILNE was one of the first cross-comparative international social network surveys of school-age adolescents’ smoking behaviour. It succeeded in describing more than 57,000 social ties and smoking in more than 11,000 adolescents from 50 schools in 6 European cities, with two name generators. The study achieved an overall participation rate of 79%, which is very satisfactory for a social network analysis.

The SILNE survey represents an important source of data for the analysis of health risk behaviours such as smoking, their association with socio-economic inequalities, and the contribution of social ties to any such association. In addition, the administration of surveys such as the SILNE survey makes it possible to study the diffusion of social norms on smoking behaviour among adolescents, the effect of tobacco control policies and their enforcement on smoking prevalence, well-being at school, and other relevant topics related to adolescent health and health promotion policies at school.

The SILNE survey provides several innovative indicators for addressing smoking inequalities. Given the limitations of previous measures of adolescent socio-economic status, particularly the limitations of the Family Affluence Scale, SILNE included a tool that measured subjective social status; SILNE also took care to assess both present and future socio-economic status, as academic performance is an indicator of adolescents’ future socio-economic status. Finally, SILNE also asked adolescents about their own disposable income, which is a possible driver of smoking behaviour alongside parental socio-economic status. Moreover, SILNE argued that social stratification operated at the school level and thus included data about the schools’ socio-economic status. Accordingly, SILNE was in a position to deliver a more fine-grained analysis of social inequalities in smoking that took into account both objective and subjective, present and future, individual and parental, adolescent and school socio-economic status.

## Additional files

Additional file 1:
**References Table** 1**.**
Additional file 2:
**Ethical information.**
