# Supplementary material for: Smoking in school-aged adolescents: design of a social network survey in six European countries
Source: BMC Res Notes. 2015 Mar 21;8:91. doi: 10.1186/s13104-015-1041-z (PMC4381513; doi:10.1186/s13104-015-1041-z)
Supplement: Additional file 1: — References Table 1. [file 13104_2015_1041_MOESM1_ESM.docx]

**Additional file 1 – references Table 1.**

**SOURCES TABLE 1**

a: Median income of country

b: Average income of the region (or province) where the city is located

c: Unemployment rate in the country

d: Unemployment rate in the city

e: Population

**Namur -Belgium:**

a: Year: 2009, <http://cytisecommunes.gedap.be>. It refers to the average of net income.

b: Year: 2009, <http://cytisecommunes.gedap.be>. It refers to the average of net income.

c: Year: 2012, Direction génerale statistique et information, SPF. http://statbel.fgov.be/fr/binaries/COMMUNIQU%C3%89%20DE%20PRESSE_EFT%202012_tcm326-218606.pdf

d: Year: 2012, Séries statisques du marché du travaille en Walonie, IWEPS, http://www.iweps.be/sites/default/files/dmc1206.pdf

e: Year: 2010, <http://cytisecommunes.gedap.be>

**Hannover - Germany:**

a: Year: 2009, Indicators and maps for spatial and urban development. INKAR. Edition 2012 Ed .: Federal Institute of Building, Urban Affairs and Spatial Development (BBSR) within the Federal Office for Building and Regional Planning (BBR [Indikatoren und Karten zur Raum- und Stadtentwicklung. INKAR. Ausgabe 2012. Hrsg.: Bundesinstitut für Bau-, Stadt- und Raumforschung (BBSR) im Bundesamt für Bauwesen und Raumordnung (BBR)]

b: Year: 2009, Indicators and maps for spatial and urban development. INKAR. Edition 2012 Ed .: Federal Institute of Building, Urban Affairs and Spatial Development (BBSR) within the Federal Office for Building and Regional Planning (BBR [Indikatoren und Karten zur Raum- und Stadtentwicklung. INKAR. Ausgabe 2012. Hrsg.: Bundesinstitut für Bau-, Stadt- und Raumforschung (BBSR) im Bundesamt für Bauwesen und Raumordnung (BBR)]

c: Year: 2009, Indicators and maps for spatial and urban development. INKAR. Edition 2012 Ed .: Federal Institute of Building, Urban Affairs and Spatial Development (BBSR) within the Federal Office for Building and Regional Planning (BBR [Indikatoren und Karten zur Raum- und Stadtentwicklung. INKAR. Ausgabe 2012. Hrsg.: Bundesinstitut für Bau-, Stadt- und Raumforschung (BBSR) im Bundesamt für Bauwesen und Raumordnung (BBR)]

d: Year: 2009, Indicators and maps for spatial and urban development. INKAR. Edition 2012 Ed .: Federal Institute of Building, Urban Affairs and Spatial Development (BBSR) within the Federal Office for Building and Regional Planning (BBR [Indikatoren und Karten zur Raum- und Stadtentwicklung. INKAR. Ausgabe 2012. Hrsg.: Bundesinstitut für Bau-, Stadt- und Raumforschung (BBSR) im Bundesamt für Bauwesen und Raumordnung (BBR)]

e: Year: 2012, Indicators and maps for spatial and urban development. INKAR. Edition 2012 Ed .: Federal Institute of Building, Urban Affairs and Spatial Development (BBSR) within the Federal Office for Building and Regional Planning (BBR [Indikatoren und Karten zur Raum- und Stadtentwicklung. INKAR. Ausgabe 2012. Hrsg.: Bundesinstitut für Bau-, Stadt- und Raumforschung (BBSR) im Bundesamt für Bauwesen und Raumordnung (BBR)]

**Tampere - Finland:**

a: Suomen virallinen tilasto [Official Statistics Finland]: Veronalaiset tulot 2011. Tulot ja kulutus 2012. [Taxable incomes in 2011. Income and consumption 2012.] <http://www.stat.fi/til/tvt/2011/tvt_2011_2012-12-19_fi.pdf>

b: Suomen virallinen tilasto [Official Statistics Finland]: Veronalaiset tulot 2011. Tulot ja kulutus 2012. [Taxable incomes in 2011. Income and consumption 2012.] <http://www.stat.fi/til/tvt/2011/tvt_2011_2012-12-19_fi.pdf>

c: Työ- ja elinkeinoministeriön Työnvälitystilasto [The Employment Service Statistics of the Ministry of Employment and the Economy]: Kuntapohjaisia vuosikeskiarvoja ELY-keskuksittain ja kunnittain keskimäärin vuonna 2011. [Annual averages by ELY centres and municipalities in 2011]. <http://www.tem.fi/files/35676/tekkun2011.pdf>

d: Työ- ja elinkeinoministeriön Työnvälitystilasto [The Employment Service Statistics of the Ministry of Employment and the Economy]: Kuntapohjaisia vuosikeskiarvoja ELY-keskuksittain ja kunnittain keskimäärin vuonna 2011. [Annual averages by ELY centres and municipalities in 2011]. <http://www.tem.fi/files/35676/tekkun2011.pdf>

e: Tampereen kaupunki [City of Tampere]: Tampereen väestö 31.12.2011. Tilastokeskuksen väestötiedot ikäryhmittäin ja osa-alueittain. [Population in Tampere in 31.12.2011. Population statistics of age groups in different parts of the city by Statistics Finland.] Tampereen kaupungin Tietotuotannon ja laadunarvioinnin julkaisusarja B 11/2012. <http://www.tampere.fi/material/attachments/v/68KzlqEtA/Tampereen_vaesto_31.12.pdf>

**Latina - Italy:**

a: Year 2009, [www.urbistat.it](http://www.urbistat.it)

b: Year 2009, [www.comuni-italiani.it](http://www.comuni-italiani.it)

c: Year 2010, ISTAT.

d: Year 2010, ISTAT.

e: Year 2010, ISTAT.

**Amersfoort - Netherlands:**

a: Statline 2012, Statistics Netherlands, The Hague (<http://statline.cbs.nl/StatWeb/publication/?DM=SLNL&PA=70843ned&D1=3-6&D2=0&D3=0,78&D4=a&HDR=G1,G2,G3&STB=T&VW=T>)

b: Statline 2012, Statistics Netherlands, The Hague (<http://statline.cbs.nl/StatWeb/publication/?DM=SLNL&PA=70843ned&D1=3-6&D2=0&D3=0,78&D4=a&HDR=G1,G2,G3&STB=T&VW=T>)

c: Statline 2012, Statistics Netherlands, The Hague <http://statline.cbs.nl/StatWeb/publication/?DM=SLNL&PA=71761ned&D1=1-3,7&D2=0,5,11,17-20&D3=74&HDR=T&STB=G2,G1&VW=T>)

d: Statline 2012, Statistics Netherlands, The Hague <http://statline.cbs.nl/StatWeb/publication/?DM=SLNL&PA=71761ned&D1=1-3,7&D2=0,5,11,17-20&D3=74&HDR=T&STB=G2,G1&VW=T>)

e: Statline 2012, Statistics Netherlands, The Hague

(<http://statline.cbs.nl/StatWeb/publication/?DM=SLNL&PA=70748NED&D1=0,2,4,16,18,20,22,24&D2=0&D3=0&D4=0,2&D5=12&HDR=T&STB=G4,G2,G1,G3&VW=T>)

**Coimbra - Portugal**

a: Year: 2010, Portuguese National Statistic.

b: Year: 2010, Portuguese National Statistic.

c: Year: 2010, Portuguese National Statistic.

d: Year: 2010, Portuguese National Statistic.

e: Year: 2010, Portuguese National Statistic.
