# Supplementary material for: Smoking in school-aged adolescents: design of a social network survey in six European countries
Source: BMC Res Notes. 2015 Mar 21;8:91. doi: 10.1186/s13104-015-1041-z (PMC4381513; doi:10.1186/s13104-015-1041-z)
Supplement: Additional file 2: — Ethical information. [file 13104_2015_1041_MOESM2_ESM.docx]

Additional file 2 - Ethical information

Italy

Name of the committee: Ethics committee, Azienda Unità Sanitaria

Locale Frosinone, Italy

Approval's reference number:: 862 , approved on 13/11/2012

 Netherlands

Name of the committee: Medical Ethical Committee of the AMC Approval's reference number: W12_256#12.17.0290

Finland
Name of the committee: Ethics Committee of the Tampere region
Favourable Statement reference number: 10/2012

Germany

Name of the committee: Ethics committee, Medical Faculty, Martin-Luther-University Halle-Wittenberg, Germany
Approval's reference number:: 2012-112, approved on 13/12/2012.

Belgium:

Name of the committee: Commission d’Éthique Biomédicale

Approval's reference number: 2012/09OCT/461

Portugal:

Name of the committee: General Directorate for Education

(Direção Geral da Educação)

Approval's reference number: Ref number 0338600001, approved

on 02/11/2012
